# Survival prediction among pathologic T4 bladder cancer patients following cytoreductive cystectomy: A retrospective single-center study

**DOI:** 10.3389/fsurg.2023.1121357

**Published:** 2023-03-22

**Authors:** Xuesong Bai, Guo Chen, Shihai Shang, Senlin Li, Huanrui Liu, Zhenwei Feng, Xin Gou

**Affiliations:** ^1^ Department of Urology, The First Affiliated Hospital of Chongqing Medical University, Chongqing, China; ^2^ Department of Urology, Wansheng People’s Hospital, Chongqing, China

**Keywords:** bladder cancer, pT4, cystectomy, age-adjusted charlson comorbidity index, prognosis

## Abstract

**Objectives:**

This retrospective study aimed to describe our institutional experience with cytoreductive cystectomy (Cx) in patients with pathological T4 (pT4) bladder cancer (BCa) and to investigate the clinicopathologic factors that can predict patient survival outcomes.

**Methods:**

We reviewed the baseline demographics, clinicopathologic features, perioperative complications, and follow-up data of 44 patients who underwent Cx for pT4 BCa at our institution between 2013 and 2021. The Kaplan–Meier curve and the log-rank test were used to analyze progression-free survival (PFS) and overall survival (OS). Univariate and multivariate analyses were performed using the Cox regression model.

**Results:**

The median age of the patients was 68 years [95% confidence interval (CI) 49–81]. Overall, 21 patients (47.7%) were estimated to have a high age-adjusted Charlson comorbidity index (ACCI) score (>4), and nine patients (20.5%) had pT4b substage BCa. None of the patients died of complications within 30–90 days after surgery. Severe complications occurred in 16% (*n* = 7) of patients within 30–90 days. During a median follow-up of 51 months, disease progression was detected in 25 patients (56.8%), and 29 patients (65.9%) died of any cause. The median PFS and OS were 15.0 and 21.0 months, respectively. The Kaplan–Meier analysis indicated that patients with high ACCI scores or pT4b BCa had worse PFS (*P* = 0.003 and *P* = 0.002, respectively) and OS (*P* = 0.016 and *P* = 0.034, respectively) than those with low ACCI scores or pT4a BCa. On multivariate analysis, pT4b substage [hazard ratio (HR), 4.166; 95% CI, 1.549–11.206; *P* = 0.005] and ACCI score >4 (HR, 2.329; 95% CI, 1.105–4.908; *P* = 0.026) remained independent risk factors for PFS and OS, respectively.

**Conclusion:**

Our study revealed that the pT4b substage is associated with a poor prognosis and that the ACCI score is a relevant and practical method to evaluate survival outcomes in patients with pT4 BCa after Cx.

## 1. Introduction

Bladder cancer (BCa) is the second most common type of genitourinary malignancy, with an estimated 573,278 new cases and 212,536 deaths globally in 2020 (1). Survival outcomes for patients with locally advanced pathological T4 (pT4) stage BCa are generally poor, with a reported 5-year cancer-specific mortality (CSM) of 81.7% (2). Moreover, these patients usually develop debilitating symptoms, such as bleeding, pain, dysuria, and urinary obstruction, leading to poor quality of life and overall worse health. Currently, the management of such patients is complex and remains a clinical challenge. According to the European Association of Urology guidelines (3), cystectomy (Cx) should be considered as the primary approach for patients with pT4a stage BCa, whereas patients with pT4b stage should receive relatively conservative multimodal treatments, such as chemotherapy, as an initial option. In patients with lesions extensively growing within the pelvic region, most minimally invasive methods rarely guarantee effective control of the primary tumor; thus, such patients may be offered cytoreductive Cx.

This approach theoretically provides the possibility to limit disease progression and improve quality of life; however, it has historically been restricted because of its high perioperative complication rates and the poor general condition of patients with locally advanced BCa. With the development of enhanced recovery programs, perioperative complications have been reduced (4), and the potential oncological benefits of cytoreductive Cx have been further investigated (2, 5–9). In this context, it is crucial to identify the appropriate traits that can contribute to predicting survival advantage to maximize its success. In this study, we retrospectively reviewed our institutional experience with Cx in patients with pT4 BCa, with a focus on progression-free survival (PFS), overall survival (OS), and complication rates, with the aim to identify the clinicopathologic features associated with prognosis.

## 2. Materials and methods

### 2.1. Patients and study design

**Figure 1:**
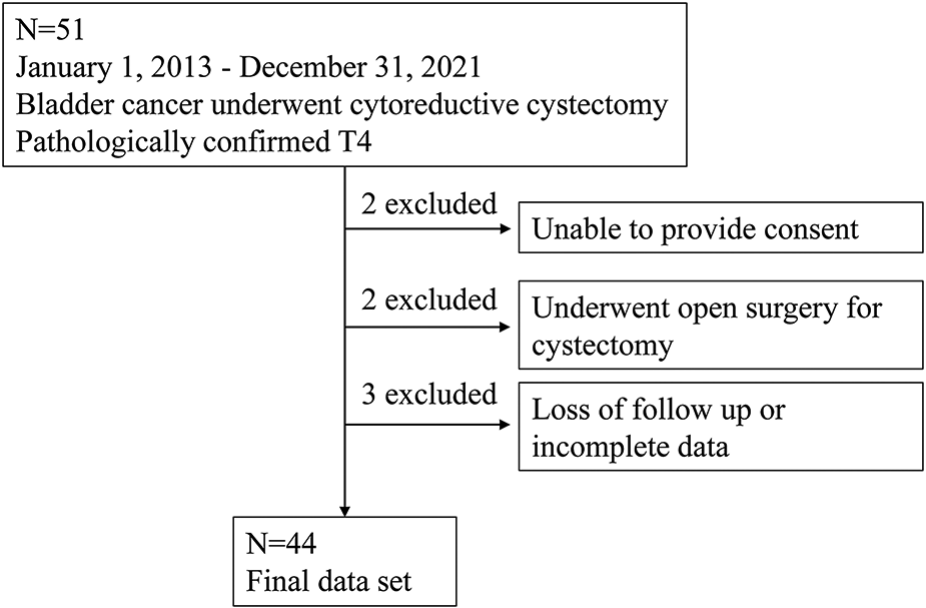
Patient selection flow diagram.

This study was approved by the Ethics Committee of the First Affiliated Hospital of Chongqing Medical University (approval no. 2022-K21; Chongqing, China), and informed consent was obtained from all patients. The patien selection flow diagram was displayed in Figure 1. A total of 44 patients undergoing radical Cx and bilateral regional lymphadenectomy at the Department of Urology of the First Affiliated Hospital of Chongqing Medical University between January 2013 and December 2021 were finally included. Hysterectomy, adjacent vaginectomy, and bilateral oophorectomy in female patients were included, and prostatectomy and seminal vesiculectomy in male patients were also included. Surgery was performed using either laparoscopic radical Cx (LRC) or robot-assisted radical Cx (RARC) strategies and through either ileal conduit or ureterocutaneostomy-based approaches, according to the personal willingness of patients and the department's expert opinions. All patients were subsequently confirmed to have pT4 stage BCa.

### 2.2. Prognosticators and outcome variables

Patient data were collected from our medical record system and included the following: age, sex, comorbidities, pre-and postsurgical treatment, information of surgery, intra- and postoperative blood transfusion, histopathology, complications, and hospital stay. The patients’ general physical condition was evaluated according to the American Society of Anesthesiologists (ASA) score (10). The age-adjusted Charlson comorbidity index (ACCI) was used to identify comorbidities (11). The Clavien–Dindo grading was utilized for postoperative classification of complication rates at 30 and 90 days after surgery (12). Grades 1 and 2 denoted minor complications, whereas grades 3, 4, and 5 corresponded to major complications. Disease progression was defined as the postoperative radiographic evidence of increasing lesions. PFS and OS were calculated since the Cx.

### 2.3. Statistical analysis

Continuous variables were summarized using the median and 95% confidence intervals (CIs), and categorical variables were summarized using frequencies and percentages. The Kaplan–Meier method and the log-rank test were used to analyze PFS and OS. Univariate and multivariate analyses were performed using the Cox regression model. Data analysis was performed using SPSS version 26.0. *P* < 0.05 was considered statistically significant.

## 3. Results

### 3.1. Baseline characteristics

Baseline clinicopathological features are shown in Table 1. The median age of the patients was 68 years (95% CI, 49–81), and 40 (90.9%) patients were males. Concerning ASA scores on preoperative evaluation, 22 (50%) patients had a score of 2, 21 (47.7%) had a score of 3, and 1 (2.3%) had a score of 4. In line with previous studies, the patient population was divided according to a cut-off ASA score of 2 for subgroup analysis (5). The median ACCI score was 4, and this value was used as a cut-off to separate patients with low and high comorbidities. A total of 23 (52.3%) patients had low ACCI scores (≤4), while 21 (47.7%) had high ACCI scores (>4).

**Table 1:** Baseline clinicopathologic features.

Sex, *n* (%)	
Female	4 (9.1)
Male	40 (90.9)
Age (years), median (interquartile range)	68 (63–74)
ASA score, *n* (%)	
2	22 (50)
3	21 (47.7)
4	1 (2.3)
ACCI class, *n* (%)	
0	2 (4.5)
1	1 (2.3)
2	10 (22.7)
3	8 (18.2)
4	2 (4.5)
5	12 (27.3)
6	6 (13.6)
7	3 (6.8)
Method of surgery, *n* (%)	
LRC	34 (77.3)
RARC	10 (22.7)
Urinary diversion, *n* (%)	
Ileal conduit	31 (70.5)
Ureterocutaneostomy	13 (29.5)
Intra- and postoperative transfusion, *n* (%)	
Negative	27 (61.4)
Positive	17 (38.6)
Pathologic T staging, *n* (%)	
T4a	35 (79.5)
T4b	9 (20.5)
Pathologic N staging, *n* (%)	
N0	35 (79.5)
N+	9 (20.5)
Clinical/Pathologic M staging, *n* (%)	
M0	40 (90.9)
M1	4 (9.1)
Histopathologic subtype, *n* (%)	
Urothelial carcinoma	36 (81.8)
Squamous cell carcinoma	6 (13.6)
Other subtypes	2 (4.5)
Lymphovascular invasion, *n* (%)	
Negative	27 (61.4)
Positive	17 (38.6)
Surgical margin, *n* (%)	
Negative	37 (84.1)
Positive	7 (15.9)
Neoadjuvant chemotherapy, *n* (%)	
Negative	40 (90.9)
Positive	4 (9.1)
Adjuvant chemotherapy, *n* (%)	
Negative	36 (81.8)
Positive	8 (18.2)

Concerning the surgical approach, 34 (77.3%) patients received LRC and 10 (22.7%) received RARC. For urinary diversion, 31 (70.5%) patients received an ileal conduit, whereas 13 (29.5%) received ureterocutaneostomy. The median duration of surgery was 262 min (95% CI, 136–419 min), and the median hospital stay was 20 days (95% CI, 9–34 days). Seventeen (38.6%) patients received blood transfusions intraoperatively or within 30 days postoperatively.

Postoperative histopathologic examination led to the confirmation of pT4a BCa in 35 patients (79.5%) and pT4b BCa in nine patients (20.5%). Lymph node metastases were found in nine patients (20.5%). Distant metastases (clinical/pathologic M1) were present in four patients (9.1%) (three lung and one retroperitoneal lymph node metastases). Neoadjuvant chemotherapy with gemcitabine/cisplatin was administered to four (9.1%) patients, while eight (18.2%) patients received adjuvant chemotherapy with gemcitabine/cisplatin or paclitaxel after surgery.

### 3.2. Survival outcomes

**Figure 2:**
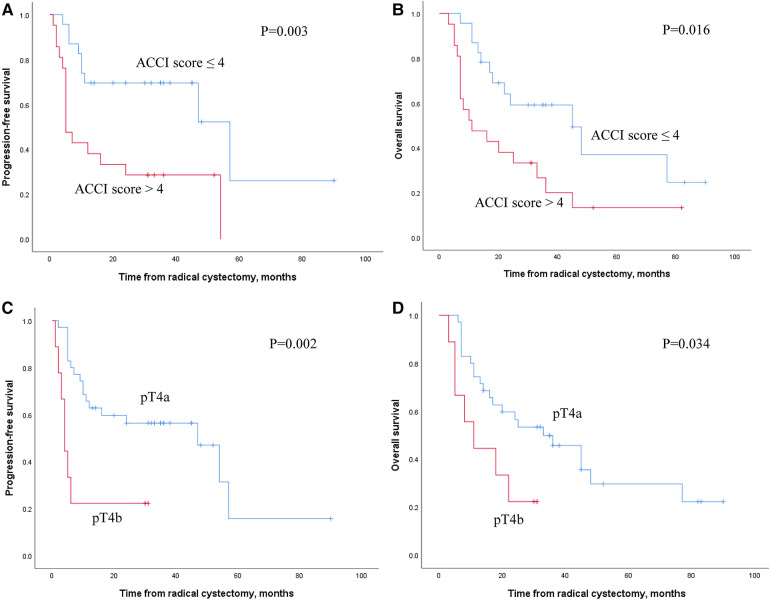
Kaplan–Meier plots for PFS and OS according to ACCI score and pT4 subgroup.

In the median follow-up of 51 months (95% CI, 15.5–104.8), 25 patients (56.8%) experienced disease progression, and 29 (65.9%) died for any reason. The median PFS and OS for all patients were 15.0 months (95% CI, 2.0–56.3) and 21.0 months (95% CI, 5.0–82.8), respectively. Kaplan–Meier analysis indicated that patients with high ACCI scores or pT4b BCa had significantly worse PFS (*P* = 0.003 and *P* = 0.002, respectively) and OS (*P* = 0.016 and *P* = 0.034, respectively) than those with low ACCI scores or pT4a BCa (Figure 2). Univariate analysis showed that ASA score >2 (HR, 2.374; 95% CI, 1.003–5.617; *P* = 0.049), ACCI score >4 (HR, 3.254; 95% CI, 1.386–7.643; *P* = 0.007), and pT4b (HR, 3.775; 95% CI, 1.516–9.399; *P* = 0.004) were significantly correlated with shorter PFS. Furthermore, ACCI score >4 (HR, 2.382; 95% CI, 1.132–5.009; *P* = 0.022) and pT4b (HR, 2.505; 95% CI, 1.027–6.109; *P* = 0.044) were significantly associated with poorer OS. In the multivariate analyses, only pT4b substage (HR, 4.166; 95% CI, 1.549–11.206; *P* = 0.005) and ACCI score >4 (HR, 2.329; 95% CI, 1.105–4.908; *P* = 0.026) remained as independent risk factors for PFS and OS, respectively (Tables 2, 3).

**Table 2:** Univariate and multivariate Cox proportional hazard regression analysis for PFS.

Variable	Univariate			Multivariate		
	HR	95% CI	*P*	HR	95% CI	*P*
Sex	0.950	0.221–4.079	0.945			
Age	1.989	0.880–4.494	0.098			
ASA score (>2 vs. ≤2)	2.374	1.003–5.617	**0** **.** **049**	2.240	0.868–5.784	0.096
ACCI class (>4 vs. ≤4)	3.254	1.386–7.643	**0** **.** **007**	2.321	0.937–5.748	0.069
Method of surgery (RARC vs. LRC)	1.424	0.555–3.655	0.462			
Urinary diversion (Ureterocutaneostomy vs. Ileal conduit)	1.816	0.793–4.158	0.158			
Intraop. transfusion	1.189	0.536–2.638	0.671			
Pathologic T staging (T4b vs. T4a)	3.775	1.516–9.399	**0** **.** **004**	4.166	1.549–11.206	**0** **.** **005**
Pathologic N staging (N+ vs. N0)	0.930	0.344–2.512	0.886			
Clinical/Pathologic M staging (M1 vs. M0)	2.107	0.619–7.169	0.233			
Lymphovascular invasion	1.010	0.444–2.298	0.981			
Surgical margin	1.146	0.387–3.392	0.805			
Neoadjuvant chemotherapy	3.115	0.904–10.729	0.072			
Adjuvant chemotherapy	0.473	0.140–1.601	0.229			

The bold values indices is *P* < 0.05.

**Table 3:** Univariate and multivariate Cox proportional hazard regression analysis for OS.

Variable	Univariate			Multivariate		
	HR	95% CI	*P*	HR	95% CI	*P*
Sex	0.515	0.152–1.751	0.288			
Age	2.090	0.994–4.392	0.052			
ASA score (>2 vs. ≤2)	2.081	0.969–4.469	0.060			
ACCI class (>4 vs. ≤4)	2.382	1.132–5.009	**0** **.** **022**	2.329	1.105–4.908	**0** **.** **026**
Method of surgery (RARC vs. LRC)	1.472	0.583–3.718	0.414			
Urinary diversion (Ureterocutaneostomy vs. Ileal conduit)	1.577	0.743–3.347	0.236			
Intraop. transfusion	1.140	0.539–2.410	0.732			
Pathologic T staging (T4b vs. T4a)	2.505	1.027–6.109	**0** **.** **044**	2.403	0.983–5.877	0.055
Pathologic N staging (N+ vs. N0)	1.043	0.423–2.570	0.927			
Clinical/Pathologic M staging (M1 vs. M0)	1.581	0.468–5.349	0.461			
Lymphovascular invasion	1.008	0.466–2.181	0.984			
Surgical margin	1.182	0.445–3.134	0.737			
Neoadjuvant chemotherapy	2.502	0.725–8.637	0.147			
Adjuvant chemotherapy	0.263	0.062–1.107	0.068			

The bold values indices is *P* < 0.05.

### 3.3. Complications

Overall, the most common complications were urinary tract infection (18%), intestinal obstruction (15%), and hemorrhagic anemia requiring blood transfusion (15%), which were mainly classified as minor complications. Within 30–90 days after Cx, 9% (*n* = 4) and 7% (*n* = 3) of patients developed severe complications (Grades 3 and 4, respectively). None of the patients died of complications within 30–90 days. The Grade 3 complications requiring invasive interventions were: injury to the deep dorsal vein of the penis, displacement of the ureteral stent, and strangulated intestinal obstruction. Four patients experienced life-threatening complications (Grade 4) due to acute renal failure requiring dialysis, postoperative bleeding, and heart failure (Table 4).

**Table 4:** Complications classified by clavien-dindo grade.

	30-day complications, *n*	90-day complications, *n*
Clavien-Dindo 1		
Renal insufficiency	3	2
Obstipation	1	
Intractable hiccup	1	
Wound infection	2	
Diarrhea	2	
Clavien-Dindo 2		
Anemia	6	
Intestinal obstruction	6	
Urinary tract infection	4	3
Anastomotic stricture		1
Pulmonary infection	1	
Clavien-Dindo 3a		
Injury of deep dorsal vein of penis	1	
Displacement of ureteral stent		1
Clavien-Dindo 3b		
Strangulated intestinal obstruction	1	
Clavien-Dindo 4		
Postoperative bleeding	1	
Acute renal failure requiring dialysis		2
Heart failure	1	

## 4. Discussion

Radical Cx has long been the gold standard for muscle-invasive BCa. In this study, we describe our single-center experience with cytoreductive Cx for patients with pT4 BCa. Patients with a high ACCI score or pT4b BCa had significantly poorer PFS and OS than those with a low ACCI score or pT4a BCa. The current guidelines do not consider Cx as a therapeutic treatment for patients with pT4b BCa. Moreover, the recent emergence of novel therapies with improved efficiency, such as immunotherapy, targeted agents, and antibody-drug conjugates therapy, have altered the treatment landscape in patients with locally advanced lesions (13). Based on our findings, we suggest that the treatment of these patients—especially those with pT4b stage BCa—should be more individualized and integrated and that cytoreductive Cx should be prudently restricted to high volume, tertiary hospitals with experience.

The benefit and safety of Cx in patients with locally advanced pT4 stage BCa have been investigated in several studies (2, 5–9). Nagele et al. (8) analyzed 20 patients with pT4 BCa subjected to primary Cx, with only two patients experiencing severe complications; after a 20-month follow-up, 55% (*n* = 11) of patients were still alive, thus supporting the rationale of primary Cx for pT4 BCa as a feasible approach with tolerable complications. Tilki et al. (9) reviewed 583 patients with pT4 bladder urothelial carcinoma treated with Cx and found that female sex, surgical margin positivity, lymphovascular invasion, lymph node metastasis, and pT4b substage were associated with recurrence-free and cancer-specific survival. In a large SEER database study by Liberman et al. (2), patients with pT4b that underwent Cx had a significantly higher CSM rate than patients with pT4a (2.1-fold) or pT3 (2.3-fold). In our study, the median OS for all patients was 21.0 months, which is longer than the previously published studies indicating 13 months (5). Our findings are partially consistent with the above conclusion, where pT4b was a predictor of poor survival; however, we failed to verify the role of the features associated with metastatic tumor dissemination. Meanwhile, several other factors, including the surgery method, urinary diversion, and intraoperative transfusion, lacked statistical significance in our Cox regression model. These limitations may have resulted from the reduced number of patients included in our analysis.

In addition to the cancer-specific variable, another significant finding from our study was that the ACCI score was strongly associated with postoperative survival outcomes, which is consistent with the previously published Cx series (14, 15). A high ACCI score has also been correlated to poor prognosis in many other types of carcinomas, such as pancreatic (16), epithelial ovarian (17), gastric (18), endometrial (19), and colorectal (20). In a study by Theresa et al. (14), by grouping patients into three categories according to the ACCI score: ≤2, 3–5, and >5, researchers found that an increased ACCI score was significantly associated with decreased OS (*P* < 0.005) but not with recurrence-free survival (*P* = 5.17) after radical Cx. It should be mentioned that our analysis chose the median ACCI score as the cut-off value to divide patients into two groups, concluding that an ACCI score >4 was an independent prognostic factor for worse OS. The high ACCI population mainly comprised elderly patients with higher comorbidity rates, often accompanied by malnutrition, inflammation, disorders of immunity and endocrine metabolism, which may increase the ability of tumor invasion and migration, resulting in increased tumor-related death. On the other hand, it should be noted that patients with high ACCI scores may die from severe comorbidities rather than BCa. Due to the lack of detailed death information records, our study was unable to further analyze this point.

In addition, our univariate analysis revealed that an ASA score >2 was significantly associated with shorter PFS. Nevertheless, this factor, which represents patients’ preoperative physical status, was not statistically relevant to any survival outcome in the multivariate Cox regression model. This contrasts with studies on elderly patients undergoing robot-assisted radical Cx that reported that the ASA score could predict OS (15). Regarding complications, in our study, no postoperative death occurred within 30 and 90 days, whereas the mortality rate reported in the curative Cx study series ranged from 1.2% to 3.2% at 30 days and from 2.3% to 8.0% at 90 days (5). However, we observed that 16% (*n* = 7) of our patients developed severe complications at 90 days after Cx, which is close to the 22% complication rate reported in a single-center study that included 1,000 patients (21).

The limitations of our study mainly resulted from its retrospective nature. First, the number of patients diagnosed with pT4 BCa selected for Cx was relatively small, even in a large-scale university hospital; thus, the limited sample size constrained the analysis of several other potentially significant factors. Second, selection bias for Cx was inevitable because surgeon backgrounds were not considered. In addition, the treatment strategies for patients with postoperative progression are so diverse that uniform evaluation is difficult. In the future, large prospective randomized trials for cytoreductive Cx are warranted to better control for these possible confounders.

In conclusion, we present our single-center experience of cytoreductive Cx for pT4 BCa, demonstrated that the ACCI score is a relevant and practical tool for evaluating postoperative survival outcomes in patients with pT4 BCa. Our findings reveal that the pT4b substage and high ACCI score are associated with poor prognosis, highlighting the need to carefully choose candidates for cytoreductive Cx.

## Data Availability

The original contributions presented in the study are included in the article/Supplementary Material, further inquiries can be directed to the corresponding author.
